# Potential demographic dividend for India, 2001 to 2061: a macro-simulation projection using the spectrum model

**DOI:** 10.1007/s43545-022-00462-0

**Published:** 2022-08-22

**Authors:** Neha Jain, Srinivas Goli

**Affiliations:** 1 Department of Economics, Indian Institute of Foreign Trade (IIFT), New Delhi, India; 2 Australia India Institute New Generation Network Scholar, The University of Western Australia (UWA), Perth, Australia; 3 Department of Fertility and Social Demography, International Institute for Population Sciences (IIPS), Mumbai, India

**Keywords:** Demographic transition, Demographic dividend, Working-age population, Health, Education, Employment, Economic Development, J10, J11

## Abstract

This paper projects potential demographic dividend for India for the period from 2001 to 2061 by using simulation modelling software, Spectrum 5.753 which integrates demographic and socio-economic changes. The simulation results highlight that a combination of favourable demographic changes and the right socio-economic policy scenario can provide a maximum demographic dividend to India. Two key findings, after checking their robustness, from the simulation modelling are: First, the effective demographic windows of opportunity for India is available for the period between 2011 and 2041, giving India roughly 30 years of demographic bonus. It is the period where the maximum of the first demographic dividend can be reaped before the ageing burden starts. Second, favourable demographic changes alone has potential to provide a demographic dividend in terms of GDP per capita over 165,000 rupees which is equivalent to an additional 43 percentage for ‘demographic-emphasis scenario’ (Rs. 548,600) compared to ‘demographic as-usual scenario’ (Rs. 382,750) in 2061. However, reaping demographic dividend is conditional on supporting socio-economic policy environment in terms of investment in human capital and decent employment opportunities.

## Introduction

India is experiencing a demographic transition that has involved changes in its population size, population growth and age distribution. There has been a rapid increase in its population from 358 million in 1950 to about 1380 million in 2020. It is expected to reach 1.7 billion by 2060 but after that, a downfall in its population size is projected. The population growth rate displays an inverted U-shaped pattern with a continuously falling population growth rate recorded since 1990–91 and projected to become negative for the period after 2060. These population changes are driven by declining fertility and mortality. The country’s infant mortality rate (IMR) has declined from 181 per 1000 live births in 1950 to 32 per 1000 live births in 2020 due to control over communicable diseases and lack of famines in the post-independence period, and it is projected to reach 5 per 1000 live births by the end of this century. Similarly, the total fertility rate (TFR) has fallen from 5.9 children per woman in 1950 to 2.2 children per woman in 2020, almost touching the replacement level fertility of 2.1 children per woman. After 2050, the TFR will stabilize at 1.7 children per woman. Further, the country witnessed a significant rise in average life expectancy at birth (LEB) from just 37 years in the post-independence period to 70 years in 2020 and projected to reach 81 years by the end of this century (Bloom et al. 2010; Bloom 2011; James 2011; James and Goli 2016; United Nations 2019).

One of the major consequences of this process of demographic transition for India is the transformation of its age-structure of the population towards the working-age group relative to the dependent group (comprising of both child and old-age population) which has opened a demographic window of opportunity for economic growth. The United Nations (2019) estimates highlight that the share of the working-age population in India has increased from approximately 58 percent in 2000 to reach a maximum of approximately 64 percent in 2035 and experiencing a downfall thereafter. Further, the population estimates suggest that the median age of an Indian population was just 29 years in 2020 while in other developed countries such as the USA, Europe and Japan, it was above 40 years (National policy for skill development and entrepreneurship report 2015), making it one of the ‘youngest large nations’ in the world. This demographic advantage can support higher economic growth for India if it is combined with the favourable policy environment of quality education, skills and health, and decent employment opportunities (Acharya 2004; Basu 2011; Bloom et al. 2003; Bloom 2011; Bhagat 2014; Chandrasekhar et al. 2006; Desai 2010; Goli and Pandey 2010; Gribble and Bremner 2012; James 2008, 2011; James and Goli 2016; Mitra and Nagarajan 2005; Navaneetham and Dharmalingam 2012).

A few studies have previously assessed the potential economic gain from fertility decline and the demographic window of opportunity for India (Aiyar and Mody 2011; Bloom and Williamson 1998; Bloom and Finlay 2008; Bloom et al. 2010; Goli and Pandey 2010; Ghosh 2016; James 2008; Joe et al. 2018 Kumar 2013; Ladusingh and Narayana 2011; Mason 2005; Mitra and Nagarajan 2005; Navaneetham 2002; Navaneetham and Dharmalingam 2012; Thakur 2012). Despite existing evidence, there is a knowledge gap on the question of potential demographic dividend for India. The above-said studies estimated the demographic windows of opportunity by considering the period before 2000 when the country had not reached a favourable demographic phase, while we have estimated and projected demographic bonus from 2001 to 2061. In this context, the main objective of this study is to present systematic documentation of economic returns as a result of demographic changes and the effective demographic window of opportunity available to India by constructing a macro-simulation model of economic growth for the period 2001–2061. Our simulation work is based on the robust theoretical framework and methodological approaches adopted by some of the earlier pioneering work in this field, such as by Coale and Hoover (1958) and later its extensions by Ashraf et al. (2013), Karra et al. (2017), and Goli et al. (2021).

This paper makes four key contributions to the existing literature: (1) We are the first one to estimate the magnitude and duration of demographic dividend for India for the period 2001–2061 by employing a computer simulation program, Spectrum 5.753, which integrates DemProj (Demographic Projection) and RAPID (Resources for the Awareness of Population Impacts on Development) modules representing demographic and socio-economic variables respectively. (2) We make demographic projections by taking 2001 as the base year in comparison to the earlier studies which have considered the period before 2000 (except for the study by Goli and Pandey 2010). Our choice of the base year is in consonance with the age-structural transition of the country which vividly depicts the onset of favourable demographic phase only after 2000. (3) We parametrize the simulation modelling along the lines of Ashraf et al. (2013) and Karra et al. (2017) by including various possible channels of demographic-economic linkages such as labour supply effect (including female labour force participation effect), human capital effect, urbanization effect, Malthus effect, and Boserup effect through which demographic transition and a consequent change in the age-structure of the population can affect economic growth. (4) The study computes demographic dividend for India by comparing GDP per capita across two demographic scenarios where no exogenous changes in the demographic factors take place to the case where the demographic variables follow the United Nations (2019) medium variant population projection. Here we assume that the country will have the right socio-economic policy environment for reaping the demographic dividend. Thus, we have presented Spectrum as a planning tool that is based on both economic theory and previous empirical evidence on macro-simulation. There is hardly any work in this direction in the Indian case. This will give a strong message to the policymakers that a combined scenario of investment in family planning, human capital, decent employment opportunities, and well-planned institutional reforms could maximize the size of potential demographic dividend for India.

The summary of the findings: First, the effective demographic windows of opportunity for India is available for the period between 2011 and 2041, giving it roughly 30 years of demographic bonus. It is the period where the maximum of the first demographic dividend can be reaped before the ageing burden starts. Second, the demographic-emphasis scenario creates a demographic dividend of over 165,000 rupees (almost an additional 43 percentage) in terms of GDP per capita by 2061 when compared to the demographic as-usual scenario, solely as a result of favourable age-structural transition and supporting socio-economic policy environment in terms of investment in human capital, family planning, decent employment opportunities, the rapid pace of urbanization, and agricultural growth.

The rest of this paper is organized as: Section “Literature review” provides a literature review. Section “Simulation strategy” deals with simulation strategy, including data inputs, assumptions, and methodology. Section “Results and discussion” discusses results and Section Conclusion concludes.

## Literature review

The role of demography on economic growth by using simulation approach has been first attempted by Coale and Hoover (1958) by making three alternative population projections (high, medium and low) for India and projected income per capita for the period 1951–1981. They assumed a constant incremental capital-output ratio and could not control for human capital and fixed factors such as land in their analysis. Their main finding was that a decline in fertility raises income per capita through the mechanism of capital accumulation (that is with low population growth, a fall in the dependency ratio positively affects the saving rate and thus investment and economic growth). After few years, the study by Enke (1971) used a dynamic demographic-economic model to compare income per capita under two different projected fertility scenarios, one of high fertility (constant gross reproduction rate) and other of low fertility (fall in the gross reproduction rate) for the period 1970–2000. It concluded that a low fertility scenario supported higher income per capita, capital stock, capital stock per worker, and a lower unemployment rate. Later studies based on simulation models included multiple productive sectors such as agriculture, industry, and services, and urbanization level (Ashraf et al. 2013). The study by Kelly (1988), however, cited many obstacles in building a credible demographic forecasts model due to general equilibrium feedbacks between demographic and economic transitions, potential changes in policy or institutions during the forecast period, and inadequate availability of data. As a result, the simulation models could not gain popularity after the mid-1980s. The Bachue demo-economic simulation models constructed for the Philippines, Kenya, Brazil, and Yugoslavia were initially designed as a planning tool (Wery and Rodgers 1980) but its review by Sanderson (1980) found their policy implications to be of less practical use for developing countries due to less focus on the supply side of the model, in particular, the neglect of input–output relationship in the agriculture sector and other technical problems.

Afterwards, the simulation models regained prominence as awareness-raising models which are designed to reflect the positive effects of fertility decline on economic growth. The study by Ashraf et al. (2013) applied a dynamic simulation model to Nigeria in which the paths of economic development were traced for two different fertility scenarios: a baseline scenario in which the fertility decline follows United Nations medium-fertility population projection and an alternative scenario where the fall in fertility follows low fertility population projection. It considered different channels by which fertility influences the economy by taking into account seven effects (Malthus, Solow, experience, dependency, childcare, child quality, and life-cycle labour supply). Their finding confirmed that a fall in fertility raises per capita income by an economically significant amount. Romero (2013) used a Computable general equilibrium (CGE) simulation model to highlight that Taiwan’s demographic transition could explain 22 percent of per capita output growth for the period 1965–2005. The National Council for Population and Development and Health Policy Project (2014) estimated demographic dividend opportunities for Kenya by considering four scenarios in the DemDiv model for the period from 2010 to 2050. It highlighted that a combined scenario of investment in education, economic policies, and family planning would raise GDP per capita by more than 12 times during the period considered. Another report by Uganda’s National Planning Authority (2014) estimated demographic dividend for the country by taking three scenarios in the DemDiv model for the period from 2010 to 2040 and found that investment in education, family planning, infrastructure, and economic reform would help Uganda to achieve a target level of GDP (USD 9500) in 2040. In recent years, several studies have projected a potential demographic dividend for Nigeria arising due to demographic changes. To mention a few, the study by Bloom et al. (2015) found that the GDP of Nigeria could be 2.7 times larger in 2030 than in 2010 due to demographic dividend and improvement in life expectancy. Karra et al. (2017) had developed a more comprehensive demographic-economic macro-simulation modelling framework for Nigeria under which the effects of child health outcomes, increase in savings, and expansion of family planning programmes were also analysed as additional channels through which fertility decline influences economic growth. The study confirmed larger economic gains resulting from fertility reduction. Lutz et al. (2019) simulated GDP per capita paths for Nigeria under three different scenarios to highlight the importance of human capital serving as a catalyst to bring the about larger impact of age-structural changes on economic growth. In the context of India, the study by Goli et al. (2021) highlighted the cumulative benefits of family planning investments from 1991 to 2061 by considering four scenarios of fertility levels. The results from the simulation exercise revealed that improvement in the quality of family planning services, and provision of quality healthcare, education, and employment opportunities could provide the maximum benefits.

In Table 1, we have summarized the estimates of the magnitude of demographic dividend and window of demographic opportunity from previous studies in the Indian context. These studies vary in their theoretical framework, methodologies adopted (such as multiple regression model, Conditional Barro Convergence Model, National Transfer Accounts Method based on life-cycle approach, etc.), empirical specification (OLS or IV), and vector of control variables. Despite these differences, a common finding from these studies is that India’s window of opportunity started much before the period 2000. The exceptions are Bloom and Finlay (2008) and Goli and Pandey (2010) who advanced that demographic windows of opportunity for the country started around 2011, and became effective from 2015. The age-structural transition of the population of India as suggested by the United Nations (2019) reveals that the country reached a favourable demographic phase at the beginning of the 2000 decade. Hence, the estimation of demographic divided after the onset of the window of opportunity assumes greater importance.

**Table 1:** Summary of the magnitude of demographic dividend and demographic windows of opportunity from previous studies in the Indian context

Study	Time frame	Magnitude of demographic dividend (in percentage)	Demographic window of opportunity	Methodology
Bloom and Williamson (1998)	1965–1990	1.34*	1.38* (estimated for 1990–2025)	Conditional Barro Convergence Model (OLS)
Navaneetham (2002)	1950–1992	Age share 15–24 = 0.30 25–49 = 0.06 50–64 = − 0.17	1980–2025	Multiple regression analysis
Mitra and Nagarajan (2005)	1950–2050	NA	1980–2035	United Nations World Population Prospects 2002 data on the relative share of the working-age group
Mason (2005)	1950–2050	0.14*	1985–2045*	National Transfer Accounts Method based on life-cycle approach (Lee and Mason Model)
James (2008)	1971–2001	24.19 (4.19)	NA	Conditional Barro Convergence Model (IV specification)
Bloom and Finlay (2008)	1965–2005	1.02	0.67 (estimated for 2005–2050)	Conditional Barro Convergence Model (OLS)
Mason et al. (2010)	1890–2100	18^#^	1990–2025	National Transfer Accounts Method based on life-cycle approach (Lee and Mason Model)
Goli and Pandey, (2010)	1951–2050	NA	2015–2040	United Nations World Population Prospects 2002 and UN’s definition for windows of opportunity
Bloom et al. (2010)	1960–2000	0.7	NA	Conditional Barro Convergence Model (2SLS)
Ladusingh and Narayana (2011)	1980–2295	9.1^#^	1980–2035	National Transfer Accounts Method based on life-cycle approach (Lee and Mason Model)
Aiyar and Mody (2011)	1961–2001	2.48 (1.03)	1970–2040	Conditional Barro Convergence Model (OLS specification)
Thakur (2012)	1981–2011	- 0.02 (0.94)	NA	Conditional Barro Convergence Model (OLS specification)
Navaneetham and Dharmalingam (2012)	1950–2040	0.4	1970–2030	Difference between the growth rates of the working-age population (25–59) and the total population
Kumar (2013)	1971–2001	2.72 (1.16)	NA	Conditional Barro Convergence Model (OLS specification)
Ghosh (2016)	1961–2011	0.3 (0.10)	1.56 (projected till 2026)	Conditional Barro Convergence Model (2SLS)
Joe et al. (2018)	1980–2010	0.45 (1.57)	NA	Conditional Barro Convergence Model (OLS specification)

NA means not available. The standard error is reported in parenthesis in column 3

^#^Income per effective consumer estimates for a first demographic dividend

*Regional estimate for South Asia. Bloom (2011, p. 11) has noted that India’s demographic indicators are similar to those of the South Asian region as a whole

## Simulation strategy

The study uses the DemProj and RAPID modules of Spectrum Suite 5.753 to estimate the magnitude and duration of demographic dividend for India by making demographic projections from 2001 to 2061. The potential demographic dividend is estimated by comparing GDP per capita across two different demographic scenarios in the DemProj module: (i) *Demographic-As-Usual Scenario*—This scenario presents a hypothetical case where the status quo continues, that is, the demographic variables in the DemProj modules are assumed to be fixed at 2001 level.^1^ (ii) *Demographic-Emphasis Scenario*—It represents a case where the demographic projections based on the United Nations (2019) medium variant fertility scenario^2^ is used for the entire period 2001–2061. Our estimation process of the demographic dividend is in line with the previous simulation exercise by National Council for Population and Development and Health Policy Project (2014), Uganda’s National Planning Authority (2014), Bloom et al. (2015), and Lutz et al. (2019).

**Fig. 1:**
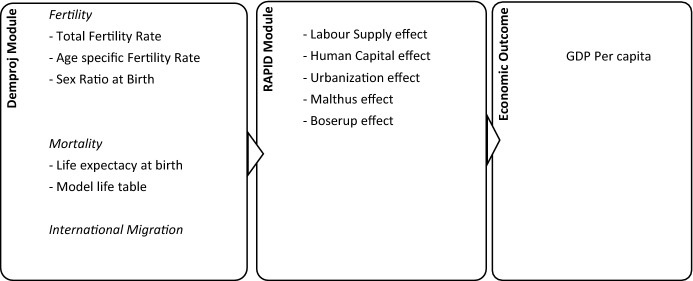
Conceptual framework in the Spectrum Suite 5.753 to link demographic-economic outcomes. Source: Authors’ framework

Since there is nothing automatic about economic returns from demographic transition unless accompanied by a favourable socio-economic policy environment in the country (Bloom et al. 2003; Bloom 2011; Mason 2005), we consider this fact by parametrizing the simulation modelling along the lines of Ashraf et al. (2013) and Karra et al. (2017) by including various possible channels of demographic-economic linkages. First, a shift in the age-structure of the population towards the working-age group will increase the potential labour supply. In addition, lower fertility induces more participation of females in the labour market. This is described in the previous literature as the *labour supply effect*. Rather than assuming full employment in our model, we allow for some degree of unemployment since everyone will not get absorbed in the market. Secondly, the accumulation of human capital, that is, investment in health, education, and skills of the population increases as lower resources are needed to be diverted for child caring and rearing (referred to in the previous literature as *human capital effect*). Third, the rapid urbanization pace is also expected to contribute to economic growth due to plenty of work opportunities, greater availability of healthcare facilities, presence of economies of scale and learning effect in industries, easier transportation, and higher participation of women in the labour market due to lower fertility rates (referred as *urbanization effect*) (Bloom et al. 2003; Bloom 2011). Next, there may be congestion of fixed factors such as land due to rising population pressure, which in turn affects per capita output (referred to as the *Malthus effect*). Lastly, population changes improve the productivity of the agricultural sector by either allowing for economies of scale or inducing technological changes (referred to as the *Boserup effect*) (Ashraf et al. 2013). The conceptual framework based on these possible channels linking demographic-economic outcomes is shown in Fig. 1. In our simulation modelling, we incorporate these channels by assuming the right socio-economic policy scenario in the RAPID module in terms of employment, economy, education, health, urbanization, and agriculture for the country. The factors which are not part of our models such as savings, capital accumulation, non-farm sector, labour market flexibility, Information and Communication Technologies use, financial market efficiency, public institutions (efficiency of governance), openness to international trade, and investment in basic infrastructure are assumed to be constant.

### Data inputs, assumptions, and methodology

Below we discuss the compilation, assumptions, and data source of each of the input indicators considered in each module in detail.^3^ While compiling inputs, we have interpolated data for some of the input indicators for which data was not available. Also, we have made standard goalpost assumptions for projection estimates to make our simulation approach more robust and reliable.

### DemProj module inputs

This module covers the following inputs which fit into the overall population processes, that is, fertility, mortality, and migration.

#### First-year population

The year 2001 is taken as the base year of the population. Its data is compiled from the 2001 census of India in single ages from 0 to 80 for both males and females which has been adjusted for the non-sampling errors.

#### Total fertility rate (TFR)

In the Demographic-As-Usual Scenario, we hypothetically assume the TFR to remain as high as 3.14 by 2061 (at the base year level). However, the TFR has already reached the replacement level fertility (TFR = 2.1) in 2020 and projected to reach 1.74 by 2060 (United Nations medium variant fertility projections 2019; National Commission on Population & Ministry of Health and Family Welfare, 2019). We assume in the Demographic-Emphasis Scenario that the trajectory of fertility in India will follow the population projections made by United Nations (2019) with an assumption of medium-fertility variant.

#### Age distribution of fertility

We assume the Age-Specific Fertility Rate (ASFR) at five years interval for the women in the age group 15–49 years to remain unchanged in the Demographic-As-Usual Scenario. However, with the significant advancement in family planning programmes and the resulting decline in the fertility level in the country, there has been a dramatic shift in the lifetime births that occur at each age group (Goli et al. 2021; National Commission on Population & Ministry of Health and Family Welfare, 2019). By using United Nations (2019) medium variant fertility projections, we observe in the Demographic-Emphasis Scenario that a majority of births are concentrated in the age group of 20–34 years for the period 2001–2020. Family planning has helped in significantly reducing adolescent pregnancies (women in the age group of 15–19). But the trends in the distribution of births for the period 2020–2061 reveals that there will be a further decline in births in the age group 20–24 also while those in the age group of 35–39 will see a moderate rise in births.

#### Sex ratio at birth (SRB)

The United Nations (2019) estimates reveal that the SRB in India in 2000 was 111 boys per hundred girls at birth due to strong-son preference and sex-selective abortion technologies (Bloom 2011; James 2011). Despite this, it is believed that rising urbanization and improved status of women with rising literacy rate can improve SRB in future (Goli et al. 2021). We assume in the Demographic-Emphasis Scenario that SRB will improve and stabilize at 108 boys per hundred girls at birth by 2061, which is in line with the projections made by United Nations (2019). The status of SRB will remain unchanged in the Demographic-As-Usual Scenario.

#### Life expectancy at birth (LEB)

The LEB has shown a continuous upward trend during 2001–2020, rising sharply for females than that of males, thereby widening the gender gap in mortality. The United Nations (2019) projections of LEB for males and females is approximately 75 years and 78 years respectively by 2061. We also consider the same LEB trends in the Demographic-Emphasis Scenario while keeping the status quo in the Demographic-As-Usual Scenario.

#### Model life tables

Model life tables are used to generate mortality schedules. We choose UN South Asia as a regional model life table for population projection. Our assumption is in line with Stover et al. (2006) and Goli et al. (2021) which found the South Asian model to be more consistent for mortality estimates for India.

#### International migration

A population projection accommodates migration as one of the three population processes. However, it can be ignored with no major effect on population projection (Spectrum Manual 2019). In the context of India, a report of the technical group on population projection (2019) assumed constant net migration due to the absence of 2011 census migration tables. The committee found no significant effect of international migration on population projection (Office of RGI and Census Commissioner 2011). Considering these facts, we assume that migration pattern by sex will follow the United Nations approach which is built-in to Spectrum and loaded by default during the projection process.

### Methodology: population projection by single ages

DemProj module makes use of the standard cohort component projection method which requires a transformation of all inputs of base year population, fertility, mortality, and migration from 5 years age groups into single ages (Spectrum Manual 2019). The Beers (1945) procedure is used to split 5 year age groups into single age groups. The formula for population projection by age and sex is as follows:

1 $${\text{Population}}_{{a,\,s,\,t,\,j}} \, = \,{\text{Population}}_{{a - 1,\,s,\,t - 1,\,j}} \, + \,0.5\, \times \,\left( {{\text{Migration}}_{{a - 1,\,s,\,t - 1,\,j}} \, + \,{\text{Migration}}_{{a,\,s,\,t,\,j}} } \right){-}{\text{Death}}_{{a,\,s,\,t - 1,\,j}}$$

where *a* refers to the age, *s* refers to the sex, *t* refers to the time and *j* refers to the scenario. Thus, the Population _*a*,* s*,* t*,* j*_ refers to the population of particular sex (*s*) at age *a* at time *t* under *j*th scenario.

Death _*a*, *s*,* t−*1,* j*_ refers to the number of deaths of particular sex (*s*) at age *a* from mid-year to mid-year under the *j*th scenario. It is calculated as

2 $${\text{Death}}_{a, \, s, \, t - 1, \, t, \, j} \, = \,{\text{Population}}_{a - 1, \, s, \, t - 1, \, j} \, + \,0.{5}\, \times \,({\text{Migration}}_{a - 1, \, s, \, t - 1, \, j} \, + \,{\text{Migration}}_{a,\,s, \, t, \, j} )\, \times \,\left( {{1}\, - \,\left( {{\text{Survival}}_{a - 1, \, a, \, s, \, t - 1, \, j} \, + \,{\text{Survival}}_{a - 1, \, a, \, s, \, t, \, j} } \right)\, \times \,0.{5}} \right)$$

where Survival_*a−*1,* a*,* s*,* t*,* j*_ indicates age-specific survival rates between age *a − *1 and *a* for the person of particular sex (*s* = male or female). Now we estimate the number of births that occurred between two mid-years. It is calculated as

3 $${\text{Birth}}_{t, \, t - 1, \, j} = \mathop \sum \limits_{a = 15}^{49} [{\text{FP}}_{a, \, t - 1, \, j} \, + \,{\text{FP}}_{a, \, t, \, j} \, \times \,0.{5}\left] {\, \times \,} \right[{\text{TFR}}_{t - 1, \, j} \, + \,{\text{TFR}}_{t. \, j} \left] {\, \times \,0.{5}\, \times \,} \right[{\text{ASFR}}_{a, \, t - 1, \, j} \, + \,{\text{ASFR}}_{a, \, t, \, j} ]\, \times \,0.{5}$$

where FP_*a*,* t*,* j*_ refers to the female population at age *a* and time *t*. ASFR_*a*,* t*,* j*_ refers to the age-specific fertility rate corresponding to age *a* at time *t* in the *j*th scenario. The number of births is multiplied by the corresponding Sex Ratio at Birth to estimate the number of female and male births. The births are then multiplied by their corresponding survival functions and the population in the age group 0–1 year is estimated as follows:

4 $${\text{Population}}_{o, \, s, \, t, \, j} \, = \,\left( {{\text{Population}}_{o, \, s, \, t - 1, \, j} \, + \,{\text{Population}}_{o, \, s, \, t, \, j} } \right)\, \times \,0.{5}\, \times \,{\text{Survival}}_{o, \, s, \, t, \, j}$$

Next, the final year population is estimated by using the same iterative procedure (that is replacing the population at the age zero and projecting for the subsequent years).

### RAPID module inputs

RAPID stands for Resources for the Awareness of Population Impacts on Development. This module covers the inputs which examine the socio-economic impacts of population growth in an economy such as labour force and new job requirements, education, health, urbanization, and agriculture. The information on these inputs is then combined with the population projections (prepared with the DemProj module) to estimate the socio-economic requisites of a country to achieve its future policy targets in a given time frame (Spectrum Manual 2019). In this module, we assume that the country will have desired socio-economic development by 2061 which can match the benchmark set by the developed countries.

#### Economy

Economy input is one of the first demographic-economic linkages representing the *labour supply effect*. An increase in the proportion of the population in the working-age group leads to the rapid growth of the labour force and thereby expansion of the economy. But this happens only if the rate of job creation is greater than the expansion of the labour force. Spectrum RAPID module covers the following economy input indicators: labour force participation rate of aged 10–14 years and 15–64 years old of both sexes, and India’s base year GDP and annual percentage growth rate of GDP. These input indicators are critical factors in determining India’s potential to realize demographic dividend (Acharya 2004; Bloom et al. 2003; Bloom 2011; Chandrasekhar et al. 2006; Desai 2010; Goli and Pandey 2010; James 2008; James and Goli 2016). The LFPR of aged 10–14 years of both sexes is obtained from different census rounds and duplicated for the inter-census period. We assume that LFPR will continue to fall and reach 0.1 percentage by 2061 under the right socio-economic policy scenario due to India’s concerted actions to eradicate child labour. The main concerning issue emerging from the recent Periodic Labour Force Survey (PLFS) rounds is a decline in LFPR among 15–64 years old of both sexes. Around half of the working-age population in India is out of the labour market. The female LFPR in India is one of the lowest in the world and less than a quarter of them were active in the labour market in 2017–18. (PLFS Annual Report 2017–18). Despite these trends, we assume that the LFPR of both males and females 15–64 years old will reach the benchmark set by the developed countries, that is, around 86 percent and 65 percent respectively, under the right socio-economic policy scenario. For GDP estimates, we take base year real GDP data from the statistics of the Reserve Bank of India (RBI). The annual real GDP growth rate of GDP during 2001–2018 is taken from the World Bank data. We target the future annual growth rate in 2061 to be 2 percentage under the right scenario, based on the GDP growth rate of large developed economies. Though GDP estimation of any nation is conditional on several economic, political, and health-related shocks (for instance, the impact of the global COVID-19 pandemic), we take a hypothetical situation where these shocks are assumed to be absent and the long term goalpost is fixed based on GDP growth patterns in developed countries. The information on economic indicators are collected from multiple sources: Ministry of Finance (MOF 2019) and Ministry of Statistics and Programme Implementation (MoSPI 2019). 

#### Education

Investments in human capital in the form of education is an important determinant of demographic dividend (Drummond et al. 2014; Lutz et al. 2019). It was one of the most essential policy interventions in East Asia which helped in its ‘economic miracle’ (Bloom et al. 2003; Bloom 2011). Spectrum software incorporates this important component of the *human capital effect* by way of several input indicators related to education such as the age of entry into school, the number of years of schooling, the school enrolment rates (in percentage), and the number of students per teacher, for both primary and secondary schools. The age of entry to primary and secondary schools in the base year (2001) was 6 and 13 years, respectively. The number of years of schooling for both primary and secondary schools is set at 5 years. The data for gross enrolment ratio (GER) and the number of students per primary and secondary school teacher is collected from the Unified District Information System for Education (U-DISE) fact sheets (NIEPA 2017). We assume that the GER for both primary and secondary schools will be 100 percent by 2061, given the current trend of remarkable progress in GER at the primary and secondary level (Economic Survey 2018–19). We further assume that the government would target to achieve developed countries benchmark of student–teacher ratios to be around 13 by 2061 in their development plans. The other information on educational indicators are also compiled from Ministry of Skill Development Entrepreneurship (MSDE 2015).

#### Health

A healthy workforce, another important component of *human capital*, is essential to realize demographic dividend (Bloom and Williamson 1998; Bloom et al. 2003; Bloom and Canning 2004; Bloom et al. 2007; Bloom and Finlay 2008; Bloom 2011; Bloom et al. 2015; Kelly and Schmidt 2005). Health input in Spectrum includes the following indicators: population per doctor, population per nurse, population per health centre, population per hospital, population per hospital bed, and annual health expenditure per person. The data for these indicators during 2001–2018 is obtained from the World Development Indicators database. In the right scenario framework, we assume that the country will achieve the health standards of developed countries by 2061.

#### Urbanization

The *Urbanization effect* is considered by including input indicators on urbanization rate (percentage), percentage of the urban population in a major city, and persons per urban household. It has been found that the level of urbanization is highly correlated with economic growth as it offers economies of scale, better employment opportunities, good education and health facilities, higher productivity, and induces lower fertility rates and, hence, higher participation of females in the labour market (Bloom et al. 2003; Bloom et al. 2008; Bloom 2011). But the experience of developing countries has shown that a rapid pace of urbanization may also result in substandard living conditions for the majority of urban dwellers. Thus, the nexus between urbanization and economic development is very complex to determine the actual impact on the living standard of urban people (Spectrum Manual 2019). India is also experiencing a fast pace of urbanization rate, expanding from 28 percentage in 2001 (Census 2001) to 34% in the year 2018 (United Nations World Urbanisation Prospects 2018). We make three key assumptions here for the goalpost 2061: first, there would be around 70 percent of the total population living in urban areas. Second, the entire urban population would be living in major cities and third, there would be 2 persons per urban household by 2061. This assumption is again based on the urbanization experience of the developed countries.

#### Agriculture

Agriculture is the main occupation sector of the Indian economy with around half of the labour force employed in it. Huge population pressure may over time reduce the amount of arable land per capita for food production in agriculture (*Malthus effect*) but the development of new technologies can boost yield (*Boserup effect*). These issues related to population pressure in the agriculture sector are addressed in the RAPID module by making standard assumptions to project the amount of arable land in agriculture, and the demand and supply of major crops (Spectrum Manual 2019).

### Methodology: GDP and GDP per capita projection

The annual rate of GDP growth is projected by assuming that GDP would increase at an exogenously specified growth rate, depending on historical trends and country-specific development plan, and not affected by the growth in population. Then GDP per capita is projected by dividing the projected GDP by the size of the population. This implies that a slower rate of population growth will lead to a rise in future GDP per capita level given constant economic growth. In other words, there will be growth in per capita income provided GDP growth is higher than the growth in population (Spectrum Manual 2019).

5 $${\text{GDP}}_{t, \, j} \, = \,{\text{GDP}}_{t - 1, \, j} \, \times \,\left( {{1}\, + \,{\text{Annual GDP growth}}_{t,j} } \right)$$

where GDP_*t*_ is the gross domestic product in time *t* under *j*th scenario.

6 $${\text{GDP per capita}}_{t, \, j} \, = \,{\text{GDP}}_{t, \, j} /{\text{Projected total population}}_{t,j}$$

where GDP per capita_*t*,* j*_ is the estimated GDP per capita in time *t* under *j*th scenario.

## Results and discussion

### Effective demographic windows of opportunity for India

**Fig. 2:**
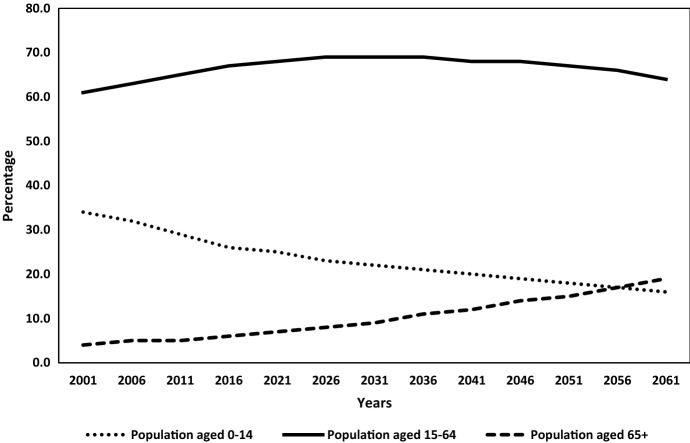
Estimated and projected demographic dividend in India, 2001–2061 Source: Authors’ framework

The United Nations defines demographic windows of opportunity as a period when the proportion of children aged less than 15 years and the proportion of the people 65 years and older fall below 30 percent and 15 percent of the population respectively. We project windows of opportunity for India using this definition by taking the year 2001 as the start of our simulation period. Figure 2 shows that India’s working-age population (aged 15–64) will increase by 8 percentage points during 2001–2036 and after that, it will start shrinking. The estimated child population (aged 0–14) will continue to fall during the entire simulation period whereas the old-age population (aged 65+) will continue to rise and after 2056, the old-age population will take over the child population in size. This pattern of demographic transition in India reveals that the proportion of children (aged less than 15 years) has reached below 30 percent of the population after 2011 and the proportion of the old-age people (65 years and older) will fall below 15 percent of the population before 2046.

**Fig. 3:**
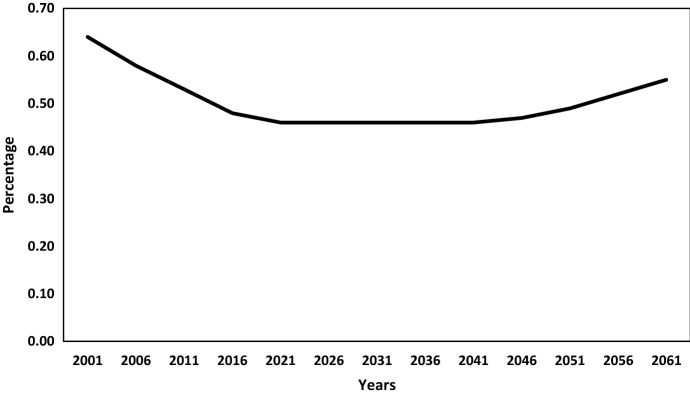
Estimated and projected dependency ratio in India, 2001–2061. Source: Authors’ estimates

But the trends in demographic dependency ratio^4^ (Fig. 3) point out that the overall dependency ratio will start rising after 2041 due to an increase in the old-age dependency, offsetting the fall in child dependency. Thus, the effective demographic windows of opportunity for India are available for the period between 2011 and 2041, giving India roughly 30 years of demographic bonus. This phase is known as ‘First Demographic Dividend’ which will give a boost to per capita income. The period after 2041, when the ageing burden starts, may provide the possibility of the ‘Second Demographic Dividend’ as the older population aids in capital accumulation from the savings done during their working years and thereby contributing to economic growth. However, it hinges on the availability of developed financial markets, a healthy older population, provision of income security and social security, which at present seems to be a formidable task in India.

Some of the previous empirical evidence on the demographic windows of opportunity for India has concluded its onset in the 1970s (Aiyar and Mody 2011; Navaneetham and Dharmalingam 2012), 1980s (Ladusingh and Narayana 2011; Mason 2005; Mitra and Nagarajan 2005; Navaneetham 2002), 1990s (Bloom and Williamson 1998; Mason et al. 2010), 2000s (Bloom and Finlay 2008) and to the latest period 2015 (Goli and Pandey 2010). Our estimates of the onset of the demographic window of opportunity for India lies somewhere in the middle of the estimates by Bloom and Finlay (2008), and Goli and Pandey (2010). Regarding the closing of the demographic window, most of the previous studies have found it somewhere around the mid-2020s (Bloom and Williamson 1998; Mason et al. 2010; Navaneetham 2002), mid-2030s (Ladusingh and Narayana 2011; Mitra and Nagarajan 2005; Navaneetham and Dharmalingam 2012) and 2040s (Aiyar and Mody 2011; Goli and Pandey 2010; Mason 2005), and 2050s (Bloom and Finlay 2008). Our finding is in line with the studies by Aiyar and Mody (2011), Goli and Pandey (2010), and Mason (2005).

### The magnitude of demographic dividend

**Fig. 4:**
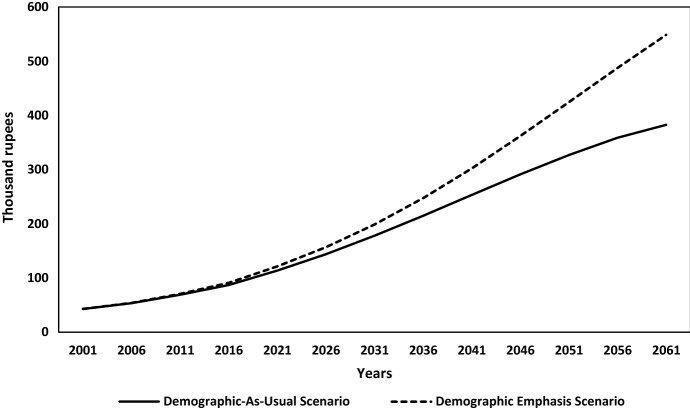
Estimated and projected real GDP per capita in India by demographic scenario, 2001–2061. Source: Authors’ estimates

The demographic dividend is reckoned when India’s possible real GDP per capita by 2061 is compared across two scenarios in the simulation modelling exercise. Under the *Demographic-As-Usual Scenario*, where the same demographic environment would continue as in the base year 2001 along with continued investment in socio-economic policies to achieve goalpost 2061, would produce a 9-time increase in real GDP per capita during 2001–2061, rising from 42,000 rupees in 2001 to about 382,000 rupees in 2061 (Fig. 4). Whereas under the *Demographic-Emphasis Scenario*, by taking demographic projections as per United Nations (2019) medium variant fertility projections along with the assumption of the right socio-economic environment in place by 2061, the real GDP per capita would go up by 12.8 times during 2001–2061, raising it to the level of 548,000 rupees in 2061. Hence, adding favourable age-structural transition (more people in the working-age group relative to dependents) to the desirable requisite socio-economic policies in terms of economy, employment, education, health, urbanization, and agriculture creates a demographic dividend of over 165,000 rupees (almost additional 43 percentage) in terms of GDP per capita by 2061.

Further in-depth analysis of India’s real GDP per capita projections due to demographic dividend for every 5 years interval shown in Table 2 highlights the rapidly rising size of potential demographic dividend if India could capitalize on its demographic transition.

**Table 2:** Projections of India’s Real GDP per capita and share of demographic dividend

Real GDP per capita (thousand rupees)			
Year	Demographic-as-usual scenario	Demographic-emphasis scenario	Demographic dividend
2001	42.55	42.55	–
2006	53.34	53.69	0.35
2011	68.84	70.45	1.61
2016	86.95	90.85	3.9
2021	113.69	121.3	7.61
2026	143.77	156.67	12.9
2031	177.79	198.63	20.84
2036	214.65	247.34	32.69
2041	253.02	302.27	49.25
2046	291.27	362.05	70.78
2051	327.02	424.68	97.66
2056	358.64	487.73	129.09
2061	382.75	548.6	165.85

*Source*: Authors’ estimates

**Fig. 5:**
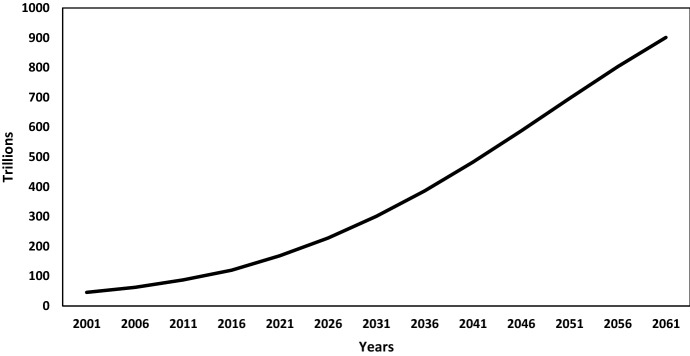
Estimated and projected real GDP in India under demographic-emphasis scenario, 2001–2061. Source: Authors’ estimates

We benchmark our GDP estimates by comparing them to the nominal GDP projections by Bloomberg Economics (Fig. 5). For the year 2025, our nominal GDP projected value of 334 trillion rupees^5^ is very close to the Figures (370 trillion rupees)^6^ reported by Bloomberg Economics (Bloomberg Economics 2019).

## Conclusion

For over two decades, economists and demographers have tried estimating the magnitude of demographic dividend for India and the demographic window of opportunity available by considering the period before 2000. However, the country’s age-structural transition reveals that India’s favourable demographic phase began only after 2000. Hence, the estimation of demographic dividend after the onset of windows of opportunity assumes greater importance. Further, our simulation modelling is capable of demonstrating that the size of our demographic dividend is conditional on the non-demographic factors by considering various demographic-economic linkages through which fertility decline and consequent changes in the age-structure of the population affect economic growth. Two main findings from our simulation exercise are: First, the effective demographic windows of opportunity for India is available for the period between 2011 and 2041, giving India roughly 30 years of demographic bonus. It is the period where the maximum of the first demographic dividend can be reaped before the ageing burden starts. Second, favourable demographic changes alone as potential to provide a demographic dividend in terms of GDP per capita over 165,000 rupees which is equivalent to an additional 43 percentage for ‘demographic-emphasis scenario’ (Rs. 548,600) compared to ‘demographic as-usual scenario’ (Rs. 382,750) in 2061. However, reaping demographic dividend is conditional on supporting socio-economic policy environment in terms of investment in human capital and decent employment opportunities.

Despite several merits, our study also has some limitations. Although we have verified robustness of our results by comparing to the findings from previous empirical studies and standard GDP projection by Bloomberg Economics, there is still scope for further improvement in our simulation modelling. The key limitations of this study are as follows: First, we could not control for life-cycle savings effect as one of the crucial channels of demographic-economic linkages due to absence of savings and capital formation input in our simulation modelling. Second, we could not allow for endogenous changes in fertility as a result of changes in income. However, Ashraf et al. (2013) found that there is no exact measurement by which fertility should respond to changes in income and this effect is found to be modest in their analysis. Third, we could not control for the impact of additional possible determinants of economic growth in our models such as the Information and Communication Technologies use, financial market efficiency, public institutions (efficiency of governance), and openness to international trade. Fourth, we could not disentangle the relative effects of diverse demographic-economic channels on economic growth which is worth investigating for future research work. Finally, we could not consider feedback effects on population growth, human capital accumulation, technological progress, and GDP growth.

Regardless of the above limitations, the findings of the study advance several implications for policy. Considering that the realization of demographic dividend is conditional on the availability of a healthy workforce, productive employment, higher education level, better infrastructure, and empowerment of women, the country as to take multi-sectoral interventions to make policy conducive to avail the demographic bonus. Currently, major hurdles in reaping the desired benefits of demographic change are dwindling spending on the education and health sector, poor quality of learning, skill mismatches, the presence of chronic illnesses and disabilities in early adulthood, falling employment rates, gender disparities (in education, health, labour market and overall sex ratio), violence, exploitation, and caste based discrimination, child marriage, falling household savings rate, urbanization of rural poverty, and rapidly rising ageing population. Also, seeing the fact that there are huge inter-state variations in socio-economic and demographic profiles, the realization of demographic dividend is conditional on the performance of northern states where the window of opportunity has just begun, and these states typically underperform in growth correlates compared to other Indian states (Bloom 2011; Economic Survey 2018–19; Goli and Pandey 2010; Goli et al. 2019; James and Goli 2016; Oxfam Indian Report 2022; PLFS Annual report 2017–18).

In terms of practical implications of the study, we have presented Spectrum-based simulation as a planning tool that makes it more reliable and transparent by including vast demographic-economic indicators, supported by economic theory and macro empirical evidence, and setting standard goalpost assumptions for the input indicators. It gives a strong message to the policymakers that when paired together, a favourable age-structure and a combined scenario of investment in health and family planning, education, decent employment opportunities, and well-planned institutional reforms and governance, could produce the best results.

## Data Availability

The data are available upon request from the authors.

## Footnotes

1 The choice of base year (2001) in Demographic-As-Usual Scenario is to match the projected and actual values of input indicators for the recent period.

2 “United Nations generally consider the medium variant to be the most likely” (Spectrum Manual 2019, p. 59).

3 It is to be noted that the inputs used in the DemProj and RAPID are inbuilt in the model, hence, leading to rigidity in determining the basis of input choice.

4 It is the ratio of the number of persons aged less than 15 years and 65 years and more to those aged 15–64 years (United Nations 2019).

5 Our real GDP estimate is converted to nominal GDP by using formula: Nominal GDP = GDP Deflator × Real GDP. We have assumed the same GDP deflator value in 2025 as it is in 2021 that is 146.10 (Source: Ministry of Statistics and Programme Implementation, MOSPI.

6 Assuming the same exchange rate between dollar and rupees in 2025 as it is in 2021, that is, one dollar = 74 rupees.
